# Management of Male Fertility in Hypogonadal Patients on Testosterone Replacement Therapy

**DOI:** 10.3390/medicina60020275

**Published:** 2024-02-05

**Authors:** Julius Fink, Hisamitsu Ide, Shigeo Horie

**Affiliations:** 1 Department of Urology, Graduate School of Medicine, Juntendo University, Tokyo 113-8421, Japan; 2 Department of Urology, Digital Therapeutics, Graduate School of Medicine, Juntendo University, Tokyo 113-8421, Japan; h.ide.me@juntendo.ac.jp; 3 Department of Urology, Advanced Informatics of Genetic Diseases, Digital Therapeutics, Graduate School of Medicine, Juntendo University, Tokyo 113-8421, Japan; shorie@juntendo.ac.jp

**Keywords:** testosterone replacement therapy, infertility, hypogonadism

## Abstract

Testosterone is crucial in regulating several body functions in men, including metabolic, sexual, and cardiovascular functions, bone and muscle mass, and mental health. Therefore, optimizing testosterone levels in men is an important step to maintaining a healthy body and mind, especially as we age. However, traditional testosterone replacement therapy has been shown to lead to male infertility, caused by negative feedback in the hypothalamic–pituitary–gonadal (HPG) axis. Recent advances in research have led to the discovery of many new methods of administration, which can have more or less suppressive effects on the HPG axis. Also, the usage of ancillary medications instead of or after testosterone administration might help maintain fertility in hypogonadal patients. The goal of this narrative review is to summarize the newest methods for optimizing fertility parameters in patients undergoing treatment for hypogonadism and to provide the necessary information for healthcare providers to make the right treatment choices.

## 1. Introduction

Testosterone replacement therapy (TRT) is gaining more and more popularity since the increase in clinical data supporting the benefits for hypogonadal patients. Cardiovascular, metabolic, and sexual functions, bone and muscle mass, and mental health can be impaired if testosterone levels are low, while testosterone replacement therapy has been shown to improve these parameters [1,2,3,4]. One large-scale study (The Massachusetts Male Aging Study) approximated the occurrence of low testosterone to be 25.3% in middle-aged/elderly men (40–70 years), whereas hypogonadism, including specific symptoms along with low testosterone levels, was observed in 6–12% of this population [5]. It is important to differentiate between primary and secondary hypogonadism, and organic (caused by the malfunctioning of the testes, hypothalamus, or pituitary gland) and functional hypogonadism (due to aging) [6]. Male hypogonadism is impairment in one or both of the two major functions of the testes: sperm production and testosterone production. These impairments are caused by diseases of the testes (primary hypogonadism) or diseases of the pituitary gland or hypothalamus (secondary hypogonadism). In general, patients with primary hypogonadism display impaired levels of testosterone and elevated levels of luteinizing hormone (LH) and follicle-stimulating hormone (FSH), while patients with secondary hypogonadism usually have reduced levels of testosterone and levels of LH and FSH on the lower end. However, chronic administration of exogenous testosterone is linked to the inhibition of the hypothalamic–pituitary–gonadal (HPG) axis, leading to impaired endogenous testosterone and sperm production, ultimately triggering the onset of male infertility [7]. Indeed, testosterone activates hypothalamic neurons, which affect gonadotropin-releasing hormone (GnRH)-secreting neurons, inhibiting GnRH secretion, the key regulator of the reproductive axis. The pulsatile secretion regulates the secretion of the gonadotropins FSH and LH, which regulate sperm and testosterone production. Due to this negative feedback in the HPG axis, exogenous testosterone has even been investigated as a potential male contraceptive therapy [8]. The severity of the shutdown also depends on the duration and dosage of testosterone, with longer treatments and higher dosages leading to more severe shutdowns of the HPG axis. Also, the administration method can influence the grade of impairment, with medium–long-acting injectable drugs being more suppressive compared with short-acting drugs [7]. Also, concomitant administration of ancillary drugs such as human chorionic gonadotropin (HCG), aromatase inhibitors (AIs), or selective estrogen receptor modulators (SERMs) can prevent or diminish the downregulated HPG axis functions in response to exogenous testosterone [9]. However, there is no consensus about the exact protocol for restoring or maintaining fertility in hypogonadal patients on TRT, and many healthcare professionals are not aware of the tools available to prevent impaired sperm production in their patients. This review will highlight novel methods to minimize fertility-related side effects due to TRT and provide directions for healthcare professionals in this field.

## 2. Exogenous Testosterone and the HPG Axis

Exogenous testosterone administration can lead to dramatic increases in serum testosterone levels. When serum testosterone levels rise, a signal to suppress the production of GnRH from the hypothalamus is sent, leading to an inhibited release of LH and FSH by the pituitary gland. As stated above, the HPG axis regulates testosterone and sperm production via the release of GnRH, consisting of LH, responsible for testosterone production in the testes via Leydig cell stimulation, and FSH, responsible for sperm production in the testes via Sertoli cell stimulation. Depending on the serum testosterone level, the HPG axis will release adequate amounts of GnRH to maintain serum testosterone levels and sperm production in balance in a self-regulating manner. Indirectly, testosterone is converted into estrogen via the aromatase enzyme. Estrogen also triggers the negative feedback in the HPG axis. As exogenous testosterone administration increases serum testosterone levels, the amount of estrogen also increases since a part of the exogenous testosterone is converted into estrogen via the aromatase enzyme. Therefore, if testosterone is administered exogenously, the hypothalamus will sense this and downregulate GnRH release, leading to impaired endogenous testosterone and sperm production in the testes. Especially if the dosage and duration of exogenous testosterone administration are significant, the downregulation of GnRH, sperm, and endogenous testosterone release will be severe. On a side note, not only testosterone but also androgenic anabolic steroids (AASs) trigger similar effects on the HPG axis. Therefore, not only TRT patients but also AAS users might face similar side effects related to male infertility. AAS users tend to use higher dosages and are, therefore, prone to harsh and long periods of HPG axis shutdown and impaired sperm production. 

## 3. Different Testosterone Products (Table 1)

(1) Long-acting drugs

(a) Injectable testosterone undecanoate

This drug is the longest-acting form of testosterone with a half-life of about 53 days. One study found that 500 mg and 1000 mg monthly injections led to almost complete suppression of LH and FSH after 16 weeks of treatment [10]. Azoospermia occurred about 12 weeks after treatment initiation in both groups. After the cessation of treatment at the 16-week mark, the sperm density reached pre-treatment levels after 20 weeks in the 1000 mg group, whereas the 500 mg group showed a steady recovery but without yet reaching initial levels [10].

**Table 1:** Summary of current TRT methods.

	Administration Method	Administration Frequency	Sperm Suppression
T undecanoate	Injection	1 time/3 months	High
T cypionate/enanthate	Injection	1–4 times/month	High
Oral T undecanoate	Oral	Daily	Low
T topical gel	Gel	Daily	Low
T nasal gel	Spray	Daily	Low
HCG	Injection	1 time/2–3 days	None
Clomiphene	Oral	Daily	None

One study showed the case of a patient in his 20s (28 years) with idiopathic hypogonadism, including oligozoospermia (2 × 10^5^ sperm/mL), a total T level of 8.73 nmol/L (with a reference value of 8.64–29.0 nmol/L), a follicle-stimulating hormone (FSH) level of 8.9 IU/L (with a reference value of 1.5–12.4 IU/L), and a luteinizing hormone (LH) level of 9.8 (with a reference value of 1.7–8.6 IU/L) [11]. After the first two injections of intramuscular testosterone undecanoate depot (Nebido^®^) separated by 6 weeks, azoospermia occurred. Spermatogenesis was recovered to 100,000 sperm/mil, 6 months after treatment cessation. At the age of 31, the patient was finally treated with Nebido^®^ for 5 years. Twelve months after termination, azoospermia was confirmed. The testosterone levels were still suppressed (6.30 nmol/L), and the LH (3.19 IU/L) and FSH (6.63 IU/L) levels were low. Thereafter, he was treated with clomiphene citrate (CC) at 50 mg/day, 5 days a week for 3 months, and his testosterone levels recovered (12.25 nmol/L), but only one mature sperm with normal motility and morphology was found in his ejaculate. One year after testosterone undecanoate cessation and numerous CC treatment periods, azoospermia was confirmed. After the cessation of CC treatment, the testosterone level decreased to 7.98 nmol/L, and the LH (7.77 IU/L) and FSH (12.22 IU/L) levels remained average [11]. Another study showed that 20 patients treated with TRT for erectile dysfunction and androgen deficiency for 4–12 months (with a median of 8 months) all recovered serum hormone levels and sperm concentrations (≥15 × 10^6^/mL) after 2–11 months (with a median of 8 months) [12]. That study also included patients using testosterone enanthate, without showing the specific data for each type of testosterone. Another study gathering data from more than 1000 healthy men with normal sperm production investigated the effects of 30 months of injectable testosterone undecanoate on fertility outcomes [13]. Participants received 500 mg/month for 30 months and sperm parameters were monitored for up to 12 months post-cessation. The median number of days to achieve a <1 × 10^6^/mL sperm concentration was 108 days. The median time for sperm production recovery was about 196 days. After 12 months post-cessation, more than 98% of the participants recovered normal sperm production [13]. These results suggest that male infertility occurs about 3 months into treatment with injectable testosterone undecanoate. Recovery of healthy spermatogenesis seems to occur about 5–6 months on average after the cessation of testosterone undecanoate injections. However, if the testosterone treatment duration is longer than 3 years, recovery might take several years and the use of ancillary drugs to stimulate gonadotropins.

(2) Medium-acting drugs

(a) Injectable testosterone enanthate

Testosterone enanthate is the most popular traditional injectable form of testosterone in Europe and many countries. It has a half-life of about 8 days. 

One study investigating the effects of intramuscular injections (1 injection per month for 3 months) of 200 mg of depot medroxyprogesterone acetate (MPA) and 250 mg of testosterone enanthate showed that the mean sperm density, total sperm count, and motile sperm count significantly decreased from the end of the second month reaching the nadir (0.7 ± 0.3 million/mL, 2.3 ± 1.4, and 0.3 ± 0.2 million, respectively) after 3 months [14]. The mean total sperm count, sperm density, and concentration of motile spermatozoa recovered to the pretreatment range after 6 months [14].

One study looking at the outcomes of intramuscular injections (1 time per week) of 50 or 100 mg of testosterone enanthate on sperm function showed that maximal suppression occurred between weeks 15 and 25 of treatment, whereas levels returned to baseline by 12 months post-termination of treatment [3]. An amount of 50 mg of testosterone enanthate per week led to severe oligozoospermia (with a concentration of <5 million/mL) in four of seven men. On the other hand, 100 mg of testosterone enanthate per week triggered azoospermia in all seven men by week 20. Sperm with normal oval morphology significantly declined in both groups in a dose-related manner before returning to initial levels at 12 months post-injection cessation [3].

Another interesting study investigating the effects of testosterone enanthate on reproductive functions divided the study into two phases: induction and maintenance [15]. The injection protocol was as follows: The induction phase included testosterone enanthate (200 mg/mL) injections twice weekly for the first 2 weeks, then weekly for 2 weeks, and once every other week for 4 weeks. This led to a harsh suppression of gonadotropins and sperm production to azoospermia or less than 100,000 sperm/mL. The maintenance phase (12 weeks) included three different schedules of injections: Injection every 3 weeks led to a partial release of gonadotropin inhibition and the reappearance of spermatozoa in the ejaculate, while plasma testosterone levels stayed within the pretreatment range. An injection every 2 weeks kept sperm production below 3 million/mL and normal plasma testosterone levels. An injection every 10 to 12 days sustained the total inhibition of luteinizing hormone and azoospermia or severe (<100,000/mL) oligospermia, while testosterone levels stayed within the normal range. Complete inhibition of LH in the presence of detectable follicle-stimulating hormone levels was crucial to trigger azoospermia. Azoospermia or oligospermia (less than 100,000/million/mL) occurred within 77.1 ± 11.6 days after the first injection. Sperm production remained suppressed throughout the observation period of 32 weeks [15]. 

One study looking at the effects of different dosages of testosterone enanthate (25, 50, 100, or 300 mg) weekly for 6 months showed that administration of testosterone enanthate to healthy men led to a significant dose-dependent parallel suppression of serum LH and FSH levels [16]. Serum LH and FSH levels were 101 ± 6% and 102 ± 3% of the control values in men in the sesame oil injection group and 91 ± 7% and 97 ± 4% of the control values in the 25 mg testosterone enanthate group, respectively. An amount of 50 mg weekly led to severe suppression of both LH and FSH levels to 65 ± 8% and 62 ± 7% of the control values, respectively. LH and FSH levels were downregulated to 32 ± 2% and 34 ± 5% in the 100 mg weekly group and 31 ± 3% and 29 ± 3% in the 300 mg weekly group, respectively [16]. Sesame oil (placebo) injections led to a reduction in sperm counts to 72 ± 7% of the control values. An amount of 25 mg weekly reduced sperm counts to 66 ± 10% of control levels, which was not significantly different from the placebo group. An amount of 50 mg per week downregulated sperm counts to 36 ± 8% compared with the control values. An amount of 100 or 300 mg per week downregulated sperm counts to 0.8 ± 0.4% and 3 ± 2% compared with the control values [16]. 

Interestingly, one study showed that a higher dose of testosterone led to less severe sperm suppression despite no difference in gonadotropin suppression [17]. The study compared the effects of high-dose and low-dose testosterone enanthate in combination with cyproterone acetate (CPA). Participants received 5 mg/day of CPA with either testosterone enanthate at 100 mg/week (CPA-5-100) or testosterone enanthate at 200 mg/week (CPA-5-200) for 16 weeks. Even though no difference in gonadotropin levels was observed between the two groups, testosterone levels were significantly higher in the CPA-5-200 group than in the CPA-5-100, and after 16 weeks, sperm counts were significantly lower in the CPA5-100 group compared with the CPA-5-200 group. Sperm counts recovered to initial levels in all subjects about 3 months after the last injection [17]. 

With testosterone enanthate, depending on the dosage and duration of TRT, sperm recovery should occur within 6–12 months after cessation, but the use of ancillary drugs and a longer treatment duration might be needed depending on each individual. 

(b) Injectable testosterone cypionate

Testosterone cypionate is very similar to enanthate and is often used interchangeably with enanthate. 

One study compared the effects of different dosages (100, 250, and 500 mg) of testosterone cypionate on gonadotropin levels and sperm production [18]. Spermatogenesis was inhibited by each of the doses, but the downregulation in sperm count was neither totally dose-dependent nor consistent among patients in the same group. LH and FSH were not detectable 2 weeks after the first injection of the 250 and 500 mg/week doses and within 5 to 6 weeks for the 100 mg/week dose [18].

One study compared the pharmacokinetics of enanthate and cypionate [19]. After an injection of 200 mg of each drug, serum testosterone, LH, and FSH levels were assessed for up to 26 days post-injection. The serum testosterone responses were similar in both groups. Serum testosterone levels increased sharply, peaking on days 1 and 2 after injection, following a gradual decrease to basal levels on day 10 (from ~3–4 mIU/mL to ~0.2 mIU/mL). Serum testosterone levels continued to decrease below basal levels on days 12 and 14 and then returned to basal levels. LH and FSH concentrations steadily decreased after injection until day 10 and then recovered to basal levels, while testosterone levels were still below initial levels [19]. 

Testosterone cypionate can be treated similarly to enanthate, with the same sperm recovery expectations.

(3) Short-acting drugs

(a) Oral testosterone undecanoate

Novel oral testosterone undecanoate preparations have been developed to be lipophilic and transported into the intestinal lymphatics while bypassing the first-pass metabolism of testosterone by the liver [20]. Since the spikes in testosterone are short (~6 h), the negative feedback in the HPG axis has been thought to be less severe compared with long-acting testosterone formulations. One recent study showed that 24 days of oral testosterone undecanoate (TLANDO^®^) led to a decrease in LH and FSH (–4.74 ± 4.92 mIU/mL and –4.91 ± 4.88 mIU/mL) levels. However, about 40% remained within the normal range from baseline [21]. This study lasted for less than 1 month; therefore, the results should be evaluated accordingly. 

One recent study investigating the effects of another oral testosterone undecanoate preparation (JATENZO^®^) compared with a topical testosterone solution (Axiron^®^) for 3–4 months showed decreases of about 70% (from ~4 to ~1 mIU/mL) from baseline in the serum concentrations of LH and FSH at the end of the intervention for both the oral and the topical testosterone versions [22]. 

Long-term studies on the fertility outcomes of oral testosterone undecanoate need to be undertaken, but based on the limited information available, it seems to be on the milder side and comparable to topical testosterone gel. 

(b) Nasal testosterone gel

One study looked at the outcomes of short-acting testosterone (a 4.5% nasal gel; Natesto^®^) on testosterone, gonadotropins, and sperm parameters for a period of 6 months [23]. An amount of 90.9% of the patients displayed normal testosterone levels of >300 ng/dL after 6 months of treatment. The FSH and LH levels stayed within normal ranges in 81.8% and 72.7% of patients after 6 months. FSH levels decreased by ~40% and LH by ~50% after 3 months. The total motile sperm count remained >5 million in 88.4% of patients after 3 months and 93.9% of patients after 6 months [23]. Another study classified hypogonadal patients into several groups depending on their baseline serum testosterone levels (<100, 150, 200, 250, and 300 ng/dL) and administered testosterone either twice- or thrice daily [24]. Every administration was 5.5 mg/nostril, with 11 mg of testosterone for each administration for 90 days. Interestingly, LH levels decreased more in patients with higher initial serum testosterone levels. LH levels decreased by approximately 1.5 IU/L in the <100 ng/dL group and 3.5 IU/L in the <300 ng/dL group [24]. 

One recent study compared the effects of nasal testosterone gel on serum testosterone, LH, FSH, and semen parameters in hypogonadal men compared with clomiphene citrate (CC) treatment [25]. FSH levels decreased by 1.2 mIU/mL but remained within the reference range. LH levels did not significantly change from baseline. The semen parameters (i.e., semen volume, sperm concentration, sperm total motility percentage, and forward progressive motility percentage) did not significantly change between the CC and nasal gel treatment groups. Moreover, 92.7% of the patients exhibited a significantly increased libido on nasal gel treatment compared with CC treatment [25]. 

One recent study looked at the direct conversion from long-acting testosterone replacement therapy to nasal gel therapy [26]. Five patients on transdermal gels, twenty patients on intramuscular testosterone cypionate, and two patients on subcutaneous pellets (with a mean age of 39 ± 8 years) for an average duration of 24.3 ± 19 months before conversion to the nasal gel treatment participated in the study. Ten out of the twenty-seven men underwent semen analyses while on long-acting TRT, showing azoospermia for each patient. FSH and LH levels remained similar from baseline to 1 month after conversion to the nasal gel treatment. Serum testosterone levels did not show any significant differences between the long-acting forms of TRT and the nasal gel. Interestingly, estradiol levels were significantly lower while using the nasal gel compared with the long-acting TRT treatments. During the 3 months on the nasal gel, all 27 patients recovered spermatogenesis, with an average sperm concentration of 50.7 million/mL [26]. Nasal testosterone gel seems to be one of the best options for hypogonadal men wanting to preserve fertility, especially in those suffering from primary hypogonadism. 

(c) Topical testosterone gel

Another trial investigating the outcomes of transdermal testosterone gel (AndroGel, 50 mg/day for 3 months) in hypogonadal men showed significant decreases in LH (from 5.05 to 3.29 mIU/mL) and FSH (from 10.32 to 6.37 mIU/mL) levels, but the LH and FSH levels remained within reference values [6]. One study compared the effects of 5, 7.5, and 10 g of testosterone gel (1%) on fertility markers [27]. Compared with initial values, LH (from about 3 to 1 IU/L) and FSH (from about 5 to 2 IU/L) levels significantly and chronically decreased for the duration of the intervention without additional improvements after 6 months. Interestingly, the decrease was more severe in the 10 g group compared with the lower-dose groups [27]. Another study showed that 6 months of 0.5–5.0 g of testosterone gel (1%) for <6 months did not affect LH or FSH levels in adolescent boys with Klinefelter syndrome or anorchia [28]. 

One study investigating the contraceptive effectiveness of topical testosterone gel vs. progestin demonstrated that 10 g of testosterone gel (1%) daily for 20 days inhibited LH and FSH to a greater extent compared with 2 or 4 mg of progestin alone [29]. Testosterone gel suppressed LH by 71%, whereas 2 mg of progestin suppressed it by 41% and 4 mg by 56%. FSH levels decreased by 52% in the testosterone gel group, and by 27% in the 2 mg and 37% in the 4 mg progestin groups [29]. 

Topical testosterone gel is also on the milder side with regard to fertility issues. However, the side effects might be dose-dependent. 

## 4. Alternatives to TRT to Raise Testosterone While Maintaining Fertility

Depending on the type of hypogonadism, there are several indirect options to restore healthy testosterone levels. These methods focus on triggering endogenous testosterone production in the testes, rather than directly administering exogenous testosterone.

(1) Human Chorionic Gonadotropin (HCG)

One recent study showed that HCG monotherapy can be efficient for men with hypogonadal symptoms and normal testosterone levels [30]. In total, 31 men (aged 25–79) experiencing hypogonadal symptoms with average testosterone levels of >300 ng/dL received varying doses of HCG (range: 1000–3000 international units (IU), twice a week). The median age of patients was 52 years old, and the mean follow-up post-HCG therapy start was 41.7 weeks, with an average HCG dose of 1529.03 IU. No major changes in testosterone (413 ng/dL to 433 ng/dL), FSH (3.1 to 3.05 mIU/mL), PSA (1.35 to 1.53 ng/mL), HCT (42.85 to 44.85%), estradiol (27.5 to 32 pg/mL), A1c (5.85 to 5.95%), or LH (4.8 to 4.0 mIU/mL) were observed [30].

HCG dosages for infertile patients usually range from 3000 to 10,000 IU, 2–3 times per week [31]. One study showed that 1500 IU of HCG administered three times/week induced spermatogenesis in 70% of men with isolated hypogonadotropic hypogonadism. Interestingly, the addition of HMG did not significantly improve testicular volume [32].

Another study conducted on 11 hypogonadotropic hypogonadal men (azoospermic) seeking fertility restoration received a single weekly HCG injection for a minimum of 12 weeks [33]. The mean baseline FSH, LH, and testosterone levels were 0.46 ± 0.28 mUI/mL, 0.39 ± 0.32 mUI/mL, and 41.3 ± 26.9 ng/dL. Spermatogenesis recovered in >90% of patients after 12 weeks. The total motile sperm count increased to 39 × 10^6^ (range: 0.0–156.9 × 10^6^), and the testosterone levels were 647.5 ± 219.0 ng/dL after 12 weeks. Moreover, an improvement in virilization, libido, and erectile function was also noted [33]. 

Another study recruited 40 hypogonadal patients and treated them with extractive HCG (n = 10 patients) for 6 months and three different types of testosterone: transdermal (n = 10 patients), undecanoate (n = 10 patients), and enanthate (n = 10 patients) [34]. After treatment, the HCG group displayed significantly higher serum concentrations of 25-OH-vitamin D and significantly lower serum concentrations of estrogens, hematocrit, PSA, and prostate volume compared with testosterone. All testosterone groups reported a significant reduction in sperm density and the percentage of spermatozoa with progressive motility, while the HCG group preserved these parameters [34]. 

One study compared the effects of CC with HCG in hypogonadal patients trying to avoid fertility-related side effects [35]. Patients were randomly assigned to one of three groups: 50 mg of CC (n = 95); 5000 IU of HCG, two times/week (n = 94); or a mix of the two drugs (CC + HCG; n = 94) for 3 months. Serum testosterone levels were upregulated from 66 ng/dL to 149 ng/dL (a 223% increase) with no major difference between the groups. Even though CC did not restore testosterone to eugonadal levels, CC seemed to be as effective as HCG in restoring testosterone levels [35].

HCG can be very effective, with no side effects on fertility, and safe for patients with secondary hypogonadism. However, HCG can be expensive or not easily available depending on the country.

(2) Human Menopausal Gonadotropin (HMG)

Treatment with LH in addition to FSH has been widely used in hypogonadotropic hypogonadism patients. Initially, FSH was made from the urine of postmenopausal women, called human menopausal gonadotropin (HMG). HMG includes LH and FSH pathways and has been on the market since the 1960s. In the meantime, a recombinant type of human FSH (rFSH) has been developed, showing pure FSH activity in contrast with HMG [36]. Even though it has some LH activity, HMG is not able to preserve the Leydig cell function and sufficient spermatogenesis. Therefore, it is often used in combination with HCG to trigger spermatogenesis in patients with hypogonadotropic hypogonadism [36]. 

One study recruited 10 men with hypogonadotropic hypogonadism and divided them into two groups: a human gonadotropins (150 IU of HMG and 1500 IU of HCG, three times/week) group and a pure FSH (150 IU, three times/week) and testosterone (250 mg of T, once/week) group [37]. Five patients first received HMG–HCG and then pure FSH in addition to testosterone, while the other five men started with pure FSH plus testosterone, for 24 months each. HMG–HCG triggered spermatogenesis after 24 months in all patients. The combination of pure FSH and testosterone did not trigger spermatogenesis, and the sperm count significantly decreased to 0.3 ± 0.1 × 10^6^/mL after 3 months and 0 after 6 months. Plasma testosterone levels increased in both treatments, but they increased significantly more after treatment with pure FSH and testosterone (35.3 ± 5.2 nmol/L) compared with HMG–HCG (20.4 ± 5.2 nmol/L; *p* < 0.05) [37]. These results support the theory that testosterone stimulation via LH is crucial for spermatogenesis, which probably cannot be achieved with FSH alone. 

One retrospective study analyzed the data of a total of 112 male patients with congenital hypogonadotropic hypogonadism [36]. A total of 70 were treated with HMG and 42 with rFSH post-HCG intervention. The initial luteinizing hormone (LH), follicle-stimulating hormone (FSH), and testosterone levels were recorded at 0.53 ± 0.77 IU/L, 0.63 ± 0.61 IU/L, and 1.10 ± 1.90 ng/dL, respectively. The HCG treatment started with a dose of 1500 IU, two times/week for 6 months. In cases where the patient remained azoospermic after 6 months, FSH (75–150 IU, two times/week) was added. Target FSH levels of 4–6 IU were reached by fine-tuning the FSH dose. The percentage of recovered spermatogenesis was similar (85.7%) in both groups, and the pregnancy rates in the hMG and rFSH groups were 38.6% (n = 27) and 51.2% (n = 21), without statistical significance [36]. 

hMG or rFSH are useful in the treatment of patients who do not respond enough to HCG treatment alone.

(3) Aromatase Inhibitors (AIs)

One study recruited patients with impaired testosterone-to-estradiol ratios and administered 100 to 200 mg of testolactone or 1 mg of anastrozole per day [38]. Seventy-four patients received 50 to 100 mg of testolactone twice daily for a mean duration of 6.0 months (ranging from 1 to 24 months), and 104 patients received 1 mg of anastrozole one time per day for an average duration of 4.7 months (ranging from 1 to 20 months). The testolactone group had an improvement in the testosterone-to-estradiol ratios. A total of 12 oligospermic men showed increased sperm concentrations (5.5 versus 11.2 million sperm per ml; *p* < 0.01), motility (14.7% versus 21.0%; *p* < 0.05), morphology (6.5% versus 12.8%; *p* = 0.05), and motility indexes (606.3 versus 1685.2 million motile sperm per ejaculate, respectively; *p* < 0.05). In the anastrozole group, similar changes in the testosterone-to-estradiol ratios were recorded (7.2 ± 0.3 versus 18.1 ± 1.0, respectively; *p* < 0.001). Twenty-five oligospermic men showed an improvement in semen volume (2.9 versus 3.5 mL; *p* < 0.05), sperm concentration (5.5 versus 15.6 million sperm per ml; *p* < 0.001), and motility index (832.8 versus 2930.8 million motile sperm per ejaculate, respectively; *p* < 0.005). Testolactone and anastrozole led to similar adaptations, including serum testosterone levels, during treatment [38].

One study recruited subfertile hypoandrogenic men with low T/estradiol (E2) ratios [39]. Patients received 1 mg of anastrozole daily. Semen was collected before treatment and at 4 months post-intervention. Three weeks into anastrozole treatment, the testosterone and bioavailable testosterone levels improved from 258.4 ng/dL and 128.8 ng/dL to 509.2 ng/dL and 297.5 ng/dL, respectively, while the estradiol levels decreased from 40.8 pg/mL to 24.6 pg/mL. The T/E2 ratio significantly improved from 6.98 to 34.5 after 4 months of treatment, and the LH levels improved from 6.41 IU/L to 10.7 IU/L and the FSH levels from 12.4 IU/L to 19.4 IU/L. Seminal volume and sperm motility did not improve. The sperm concentration significantly improved from 4.7 × 10^6^/mL to 13.1 × 10^6^/mL and the total motile count from 4.6 × 10^6^ to 8.0 × 10^6^ [39].

In another study, ten male patients with idiopathic hypogonadotropic hypogonadism (IHH) received anastrozole (1 mg/d orally) [40]. After 2 weeks of treatment with anastrozole, the testosterone, LH, and estradiol levels had recovered to be within the normal range [40]. 

AI monotherapy in hypogonadal oligo-azoospermic patients is still not completely understood to this day, but the results look promising.

(4) Selective Estrogen Receptor Modulators (SERMs)

Clomiphene has been shown to be effective in elevating testosterone levels in hypogonadal men. However, testosterone therapy seems to be superior in increasing testosterone and treating hypogonadal symptoms [41]. One meta-analysis showed that clomiphene citrate is a potent drug for treating both the biochemical as well as clinical symptoms of hypogonadal patients, with few reported side effects and good safety aspects [42].

One study showed that clomiphene increased testosterone from 235 to 438 ng/dL (*p* < 0.05) and testosterone treatment from 231 to 541 ng/dL (*p* < 0.05) [41]. Unfortunately, we could not gather information about the exact clomiphene and testosterone dosages used in that study. Interestingly, the changes in estradiol levels were similar between both groups (3.5 ng/dL vs. 3.6 ng/dL; *p* = 0.87). Both groups showed improvements in hypogonadal symptoms using the ADAM (CC: 3.5 vs. 1.5; TST: 5.0 vs. 2.0; *p* < 0.05) and qADAM questionnaire (CC: 30.7–32.5; TST: 32–36; *p* < 0.05), but men on testosterone showed greater symptom resolution [41].

One study conducted on 29 patients with an initial total testosterone level of 228 ± 48 ng/dL treated for > 3 years with clomiphene (patients were started on 25 mg every other day and were titrated to 50 mg every other day depending on their serum T level) showed increases in testosterone to a mean of 582 ± 227 ng/dL (*p* < 0.001) and improvements in the ADAM questionnaire scores (*p* = 0.01) [43]. After 36 months, 75% of the patients stayed at 25 mg every other day. LH levels increased from 2.0 to 8.6 after 1 year, 7.2 after 2 years, and 8.2 IU/mL after 3 years [43]. Taylor and Levine also showed that compared with testosterone gel therapy, clomiphene treatment led to similar improvements in testosterone levels and ADAM questionnaire scores at a lower cost. No side effects were recorded. Moreover, patients on clomiphene treatment did not show increases in hemoglobin, PSA, or cholesterol levels [44]. Another study showed that quantitative ADAM scores were similar in patients treated with clomiphene compared with those treated with testosterone (gel or injection) [45]. Moreover, testosterone levels while on clomiphene reached physiologic levels. 

Tamoxifen has been shown to improve testosterone, gonadotropin, and sperm release, making it an alternative treatment option for testosterone deficiency in men. 

Clomiphene is made of zuclomiphene and enclomiphene. One study investigated the effects of oral enclomiphene citrate on the endogenous production of testosterone and sperm in hypogonadal men [46]. After 6 months of 25 mg/day, the total testosterone levels were 525 pg/dL while demonstrating increased LH (about 1.8 to 6 mIU/mL) and FSH (about 1 to 5.5 mIU/mL) levels. Enclomiphene citrate increased sperm counts > 75 million/mL in all patients after 3 months [46]. 

Tamoxifen treatment in patients with idiopathic oligozoospermia significantly increased testosterone, while the average sperm concentration and total sperm improved by about 70% after an average intervention duration of 5.5 +/− 0.4 months [47]. No major difference was observed between 5 and 10 mg once daily or at a higher dose of tamoxifen (10 mg twice daily) with regard to basal or LHRH-stimulated gonadotropin and testosterone response or the E2/T ratio, sperm density, and total sperm output. However, in both groups, the sperm motility and morphology did not change [47]. 

However, the side effects from tamoxifen seem to be harsher than those from clomiphene, including gastrointestinal distress, venous thromboembolic events, and other cardiovascular events, making tamoxifen a suboptimal choice for the treatment of testosterone deficiency [48].

SERMs, especially clomiphene might be a good alternative to testosterone in hypogonadal men seeking fertility. 

## 5. Ancillary Drugs during TRT to Maintain Fertility

TRT can rapidly trigger infertility; therefore, several ancillary drugs can be prescribed to prevent those negative side effects on fertility parameters during TRT. These ancillary drugs stimulate endogenous testosterone production and therefore help prevent a complete shutdown of the HPG axis. Note that many of these drugs can be administered on their own as alternatives to TRT, as seen in the previous section.

(1) Human Chorionic Gonadotropin (HCG)

Human chorionic gonadotropin can stimulate endogenous testosterone production in patients with secondary hypogonadism since it mimics the effects of LH and binds to the same receptors. HCG was originally marketed for the treatment of female infertility via the stimulation of follicular maturation and the progression of immature oocytes. However, due to its powerful testosterone-releasing effects without side effects on fertility, the use of HCG in men has been on the rise in recent years.

HCG was first recorded in the blood and urine of pregnant women in 1927, with the belief that it was released from the anterior pituitary. It was shown that subcutaneous injections of HCG into intact immature female mice triggered follicular maturation, luteinization, and hemorrhage into the ovarian stroma. Thereafter, it was shown that HCG is not released from the pituitary gland, but from the chorionic villi of the placenta. HCG treatment in men has been investigated since the 1950s and has been demonstrated to improve endogenous testosterone levels [49].

High intratesticular testosterone (ITT) levels, usually higher than serum testosterone levels, seem to be necessary for healthy spermatogenesis in men, even though the quantitative relationship between ITT and sperm production is not understood to this day. One study recruited 29 men with normal reproductive function and administered 200 mg/week of testosterone enanthate in addition to either a saline placebo or 125, 250, or 500 IU of HCG every other day for 3 weeks [9]. The baseline serum testosterone level (14.1 nmol/L) was only 1.2% of the ITT level (1174 nmol/L). After testosterone administration, LH and FSH were inhibited to 5% and 3% of baseline, respectively, and ITT was downregulated by 94% (1234 to 72 nmol/L) in the testosterone/placebo group. Interestingly, ITT increased in a dose-dependent manner with increasing HCG doses, 25% less than the initial value in the 125 IU HCG group, 7% less than the initial value in the 250 IU HCG group, and 26% greater than the initial value in the 500 IU HCG group. This shows that concomitant low-dose HCG treatment can maintain ITT in a healthy range in men with testosterone-induced gonadotropin suppression [9]. 

One study investigated whether normal FSH levels are required to trigger spermatogenesis after chronic gonadotropin and testicular suppression [50]. After a 3-month control period, patients were administered 200 mg/week of testosterone enanthate to inhibit LH and FSH for a total of 9 months and until successive sperm concentrations (performed twice monthly) showed azoospermia or severe oligozoospermia (mean sperm concentrations of <3 × 10^6^ spermatozoa/mL) for 6 months. Thereafter, while on TRT, all patients were administered 5000 IU of HCG three times/week for 6 months, leading to a replacement of LH while leaving FSH inhibited. Testosterone administration led to severe inhibition of sperm concentrations from 79 ± 7 × 10^6^ spermatozoa/mL (mean ± SEM) during the control period to 0.8 ± 0.5 × 10^6^/mL after 12 weeks of testosterone treatment. After the start of HCG treatment in addition to TRT, sperm concentrations significantly improved in all patients, attaining a mean of 24 ± 4 × 10^6^ spermatozoa/mL after 12 weeks. During the 9 months of TRT and 6 months of additional HCG treatment, serum FSH was inhibited from about 120 ng/mL to undetectable levels of <25 ng/mL after 1 month of TRT. Urinary FSH excretion was markedly inhibited to levels found in prepubertal children and adults with hypogonadotropic hypogonadism during the TRT + HCG treatment period. Sperm production can be reinitiated by HCG after chronic gonadotropin and testicular inhibition, even though FSH levels are low. Average levels of FSH are not necessary for sperm production reactivation in gonadotropin-suppressed patients [50]. Testosterone activates the androgen receptors (ARs) in Sertoli cells, triggering spermatogenesis [51]. FSH seems to act both independently and with testosterone to activate Sertoli cell proliferation, leading to spermatid maturation [52]. In rodents, it has been shown that a lack of FSH or FSHR does not lead to sterility, but rather to a downregulation in testis size, probably due to the reduced Sertoli cell number and capacity to support and nurture germ cells [1,53]. 

One study showed that out of 26 men treated with TRT (19 on injectable and 7 on transdermal testosterone) and intramuscular administration of 500 IU of HCG every other day, no patient became azoospermic [54]. Testosterone levels were 207.2 vs. 1055.5 ng/dL (*p* < 0.0001), and free testosterone levels were 8.1 vs. 20.4 pg/mL (*p* = 0.02). Sperm parameters did not change for more than a 1-year follow-up period, during which nine patients had a successful pregnancy with their partner [54]. 

Concomitant HCG treatment seems to be effective in maintaining fertility in men on TRT. However, HCG alone probably triggers similar increases in serum testosterone levels. Therefore, if HCG is available, HCG alone should be used first, and the addition of testosterone should only be considered if serum testosterone does not increase to the target level. In this case, the addition of testosterone would negatively impact semen parameters. 

Selective Estrogen Receptor Modulators (SERMs)

SERMs can also be used in patients with secondary hypogonadism. However, SERMs indirectly increase GnRH release via the inhibition of estrogen receptors. In male patients, even though promising results have been published, the usage of SERMs remains off-label. The main SERMs used for fertility issues are clomiphene and tamoxifen citrate. These drugs indirectly trigger the release of FSH and LH by inhibiting estrogen receptors in the hypothalamus and pituitary gland, resulting in an increase in gonadotropin-releasing hormones, ultimately stimulating the Leydig cells to release testosterone and the Sertoli cells to increase spermatogenesis [7]. 

Clomiphene works by downregulating estrogen’s negative feedback loop in the hypothalamus. This triggers gonadotropin release and improves testicular function. Tamoxifen works similarly to clomiphene, but unlike clomiphene, tamoxifen is very active in the periphery, leading to its usage in treating hormone-sensitive breast cancer. On the other hand, it has also been shown to be a powerful treatment for early-onset gynecomastia in men. But most interestingly, it has been shown to increase gonadotropins and improve sperm parameters in sub-fertile men [49]. 

One meta-analysis showed that SERM use correlates with an improved pregnancy rate (pooled OR: 2.42; 95% CI: 1.47–3.94; *p*= 0.0004), sperm concentration (WMD: 5.24; 95% CI: 2.12, 88.37; *p* = 0.001), sperm motility (WMD: 4.55; 95% CI: 0.73, 8.37; *p* = 0.03), FSH (WMD: 4.19; 95% CI: 2.05, 6.34; *p* = 0.0001), and testosterone (WMD: 54.59; 95% CI: 15.92, 93.27; *p* = 0.006). When looking at different dosages and different SERMs, the subgroup analysis demonstrated that both 50 mg of clomiphene and 20–30 mg of tamoxifen daily lead to significant improvements compared with the 25 mg dosage of clomiphene [7].

One study assigned oligozoospermic patients to two treatment groups: (1) 20 mg/d of tamoxifen citrate and 120 mg/d of testosterone undecanoate (n = 106), and (2) placebo treatment (n = 106) for 6 months [2]. In the tamoxifen/T group, the total sperm count increased from a median [25th, 75th percentile] of 27.1 × 10^6^ cells/mL [9.4, 54.0 × 10^6^ cells/mL] to 61.5 × 10^6^ cells/mL [28.2, 119.6 × 10^6^ cells/mL], the progressive motility increased from 29.7% ± 12.0% to 41.6% ± 13.1%, and the normal morphology increased from 41.2% ± 14.0% to 56.6% ± 11.5% after 6 months. The spontaneous pregnancy rate was 33.9% in the tamoxifen/T group and 10.3% in the placebo group [2]. This method of concomitant administration of testosterone and SERMs might be effective in maintaining fertility in men on TRT. However, many clinicians are not aware of the benefits and might be reluctant to administer such a treatment method. Serum testosterone levels should be closely monitored since the addition of a SERM can increase testosterone even more.

(2) Aromatase inhibitors (AIs)

Aromatase inhibitors have been prescribed to treat male infertility for a long time, but there is no consensus about the efficacy and safety of AIs in the treatment of male infertility to this day. Estradiol, mainly produced through the aromatization of circulating testosterone in adipose tissue in men, affects the functions of gonadal axis regulation and spermatogenesis. Increased estradiol levels enhance the feedback in the hypothalamic–pituitary axis, triggering decreases in LH, FSH, and testosterone production. Aromatase inhibitors are traditionally used for the treatment of metastatic breast cancer, but they are also used in hypogonadal men by altering the T/E2 ratio and inhibiting increases in estradiol. Several AIs have been studied in off-label trials, with the most commonly tested steroidal AIs being testolactone and exemestane, and the most commonly tested nonsteroidal AIs being anastrozole and letrozole [55]. Furthermore, aromatase inhibitors can be classified as (1) nonselective, such as aminoglutethimide and testolactone, or (2) selective, such as anastrozole (Arimidex), letrozole (Femara), exemestane (Aromasin), vorozole (Rivizor), formestane (Lentaron), and fadrozole (Afema). Moreover, according to the level of selectivity, AIs can be classified into three generations, with the third generation having the highest selectivity and potency. (1) The first generation includes compounds such as aminoglutethimide (nonsteroidal). (2) The second generation includes compounds such as fadrozole (nonsteroidal) and formestane (steroidal). (3) The third generation includes compounds such as anastrozole (nonsteroidal), exemestane (steroidal), and letrozole (nonsteroidal). Compared with first-generation AIs, second-generation aromatase inhibitors are twice as efficient, whereas third-generation compounds are three to four times more efficient.

AIs are mainly used to control elevated estradiol (E2) levels to prevent adverse effects in men on testosterone replacement therapy. One study examined the impact of anastrozole in men with elevated E2 levels due to testosterone therapy [56]. Patients with E2 levels of >60 pg/mL, regardless of the symptoms, or 40–60 pg/mL with subjective symptoms received 0.5 mg of anastrozole three times/week. The median pre-treatment E2 levels were 65 pg/mL vs. the 22 pg/mL post-treatment E2 levels. The testosterone levels did not show significant changes from pre- (616 ng/dL) to post-treatment (596 ng/dL) [56]. AIs are an effective way to control E2 levels during TRT. However, the dosage needed to maintain E2 levels within the optimal range depends on each individual and requires close monitoring by a healthcare professional. 

## 6. Ancillary Drugs after TRT to Restore Fertility

Generally, patients are not aware of the long-term repercussions of TRT with regard to spermatogenesis. Several months or years after initiation of TRT, patients might want to have kids. In this case, it is crucial to understand which drugs can restore endogenous testosterone and sperm production. Again, the majority of these drugs can also be used as an alternative to TRT or as an addition to maintain fertility while on TRT.

(1) HCG/FSH

One meta-analysis about testosterone as a short-term hormonal contraceptive in eugonadal men showed that the mean probability of sperm recovery to 20 million sperm/mL was 67% within 6 months, 90% within 12 months, 96% within 16 months, and 100% within 24 months [57]. However, older men who start with a low–normal sperm count might need more time to recover [57].

One retrospective study on the effects of HCG therapy on the recovery of spermatogenesis after testosterone recruited 49 men (16 (32.7%) used injectable testosterone, 16 (32.7%) transdermal testosterone, 7 (14.3%) pellets, 6 (12.2%) a combination of the three, and 4 (8.2%) unknown types of testosterone), who were given 3000 units of HCG every other day after termination of testosterone treatment [58]. In addition to HCG, 35 (71.4%) used clomiphene citrate, 28 (57.1%) tamoxifen, 10 (20.4%) anastrozole, and 1 (2.0%) recombinant FSH. The mean baseline FSH, LH, and testosterone levels before termination of testosterone therapy were 1.39 mIU, 0.85 mIU, and 573.5 ng/dL, respectively. A return of spermatogenesis or an improvement in sperm density to >1 million/mL occurred in 47 (95.9%) men after the initiation of HCG therapy. The average time to recover sperm (for azoospermic men) or increase sperm to > 1 million/mL (for severely oligospermic men) was 4.6 months. The mean first sperm density was 22.6 million/mL, with average FSH levels of 4.04 mIU (an increase of 191% vs. baseline), average LH levels of 3.12 mIU (an increase of 267% vs. baseline), and average testosterone levels of 475.8 ng/dL (a decrease of 17.0% vs. baseline) [58].

One study looked at the effects of HCG combined with recombinant human FSH (rhFSH) on sperm parameters in infertile men with adult-onset idiopathic hypogonadotropic hypogonadism [59]. Patients received 150 IU of rhFSH two times per week and 5000 IU of HCG two times per week. After 6–12 months of treatment, all patients could ejaculate, and spermatogenesis was recovered in 71% of the patients [59]. Unfortunately, post-treatment LH and FSH levels were not measured in that study. 

One study showed that older age and a longer duration of TRT decrease the chances of sperm recovery (based on the criterion of a total motile count of 5 million sperm) at 6 and 12 months in testosterone-induced infertile men [60]. An amount of 3000 IU of HCG was administered three times per week. All men in that study were also prescribed either clomiphene citrate or tamoxifen citrate. The average age of the patients was 40.2 years, and the median duration of TRT was 2 years, and the method of testosterone administration was as follows: Thirty-five men used injectable testosterone, twenty-two used topical testosterone, and nine used pellets. Forty-six men (69.7%) reached a total motile count of >5 million within 12 months, at a mean age of 38.3 years after a median duration of TRT of 1.67 years. For patients who could not recover spermatogenesis, the mean age was 44 years with a median duration of TRT of 4.0 years. FSH levels increased from 0.47 to 1.59 after 6 months and to 2.6 IU/L after 12 months, and LH levels from 0.2 to 1.17 after 6 months and 1.47 IU/L after 12 months of HCG therapy [60]. 

Interestingly, one study documented that single injections of 400, 2000, and 4000 IU of HCG resulted in significant testosterone level increases in hypogonadal as well as eugonadal males without differences between the dosages [61]. In hypogonadal patients, 400, 2000, and 4000 IU of HCG increased testosterone levels from about 200 to 400 ng/dL, whereas in eugonadal patients, 400, 2000, and 4000 IU of HCG resulted in an increase from about 450 to 700 ng/dL in serum testosterone levels [61]. Higher doses of HCG did not increase testosterone levels to a greater extent compared with low HCG doses [61]. 

HCG treatment is effective in restoring spermatogenesis after TRT. In some cases, the addition of HMG or rFSH might be necessary. We recommend considering the addition of HMG or rFSH if HCG treatment has not led to the desired sperm parameter improvements after 3 months.

(2) SERMs

(a) Clomiphene citrate (CC)

One case study recruited a 29-year-old individual showing symptoms of impotence and low libido for one year [4]. He used anabolic steroids for 8 months (January–August 1992), alternating 16-week cycles of testosterone cypionate (DepoTestosterone) at 1500 to 1800 mg per week, and oxymetholone (Anadrol) at 560 mg per week. After stopping these anabolic agents in August 1992, he became impotent, suffering from a lack of spontaneous erections and low libido. He used an unknown dose of HCG for 4 weeks in September 1992 without seeing any improvements in libido or potency. In July 1993, one year after his steroid use, he displayed a reduced testicular volume of 10 mL on both sides and 2 cm of gynecomastia on both sides. His serum gonadotropin and free testosterone levels were low, (0.6 mIU per ml for FSH, 1.9 mIU per ml for LH, and 7.1 pg per mL for free testosterone).

After a month of therapy with clomiphene, 50 mg orally per day, no improvement in potency or libido occurred, but morning erections started. FSH, LH, and free testosterone levels improved slightly, but not within the normal range. One month after receiving 100 mg/day, improvements in libido and potency occurred [4]. 

Another similar case study showed the case of a 30-year-old male who abused several different types of anabolic steroids [62]. The patient suffered from low libido and depression. Blood tests showed a total testosterone level of 71 ng/dL (reference range: 260–1000 ng/dL), a free T level of 29 pg/dL (reference range: 34–194 pg/dL), an LH level of 3.7 mIU/mL (reference range: 1.5–9.3 mIU/mL), and an FSH level of 2.4 mIU/mL (reference range: 1.4–18.1 mIU/mL). He started treatment with 100 mg of clomiphene citrate for 5 days, leading to a total testosterone level of 828 ng/dL 2 weeks later. The patient noticed improvements in mood, libido, and energy. Two months after the five days of clomiphene citrate administration, the symptoms came back, and his total testosterone level decreased again to 301 ng/dL. Thereafter, he continued treatment with clomiphene citrate for 2 months, leading to a total testosterone level of 705 ng/dL and an LH level of 26.3 mIU/L, and his symptoms resolved [62].

CC is a potent treatment option for spermatogenesis recovery after TRT. Concomitant use of HCG might improve the effects and recovery time. 

Besides the above-mentioned traditional methods, there are also several other methods, such as pulsatile GnRH administration, dopamine receptor agonists, and the most commonly used male fertility treatment technique, assisted reproductive technology.

Pulsatile GnRH administration has been shown to be similarly effective in restoring LH, FSH, and testosterone levels compared with HCG treatment [63]. However, this method might not be practical nor economically feasible for many patients since similar results can be achieved with HCG treatment. 

In case infertility is due to elevated prolactin levels, dopamine receptor agonists such as cabergoline can be effective in treating this condition [8].

In case all the aforementioned methods do not work, there is still the option to try assisted reproductive technology, which can lead to great results, even with impaired fertility parameters.

## 7. Conclusions

**Figure 1:**
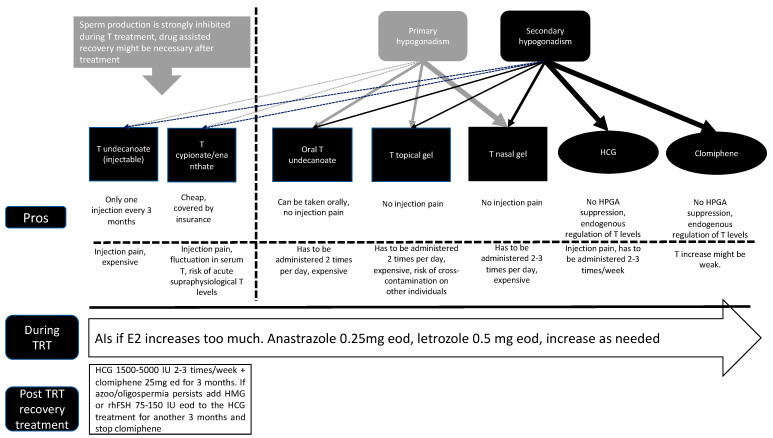
Proposed flow chart of treatment options for hypogonadal men. TRT: testosterone replacement therapy, T: testosterone, and HCG: human chorionic gonadotropin.

**Figure 2:**
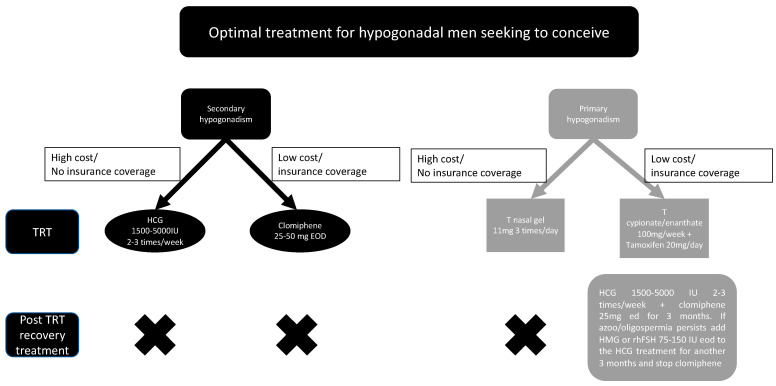
Proposed treatment options for hypogonadal men seeking TRT and fertility. TRT: testosterone replacement therapy, T: testosterone, and HCG: human chorionic gonadotropin.

The preservation of fertility is one of the major concerns for men seeking testosterone replacement therapy. However, nowadays, due to the development of novel testosterone administration methods, the suppressive effects of testosterone treatment seem to be manageable, especially with short-acting testosterone preparations. However, the lack of long-term studies prevents any clear conclusions yet. As a take-home message for clinicians, for patients seeking to conceive and who are responding to HCG or clomiphene treatment, the use of these drugs might be a better option compared with testosterone. However, long-term studies on HCG or clomiphene treatment of >3 years in men are lacking. Concomitant use of HCG or clomiphene during TRT might not be optimal in men seeking fertility; therefore, monotherapy of HCG or clomiphene should be explored first. The use of ancillary drugs such as SERMs, HCG, HMG, and AIs seems to be an effective way to recover endogenous testosterone and sperm production after testosterone treatment. The duration of fertility recovery seems to be dependent on the dosage and length of the testosterone treatment. Physicians treating hypogonadism should be aware of all these options and make the right decision based on the needs and conditions of each patient. As a side note, it is not completely understood how androgens affect the physiology of the sexual accessory glands. This could also represent a link between hypogonadism and infertility [64]. Figure 1 represents a proposed flow chart for treatment options for hypogonadal men. Figure 2 represents a proposed treatment option for hypogonadal men seeking TRT and fertility.

## 8. Comments

With regard to the clinical considerations of TRT options for men seeking to maintain their fertility, as shown in Figure 2, depending on the country and the availability of HCG and clomiphene for secondary hypogonadism, these options should be explored first. Clomiphene is the cheaper and more readily available drug, but HCG might be more effective in some cases. The high effectiveness of HCG has to be weighed against the high costs and administration method discomfort (injections) compared with clomiphene when selecting a treatment method. In some cases, concomitant use of both drugs can lead to superior results. As for primary hypogonadism, the nasal spray option seems to lead to solid improvements in serum testosterone levels while minimizing side effects on spermatogenesis. The downsides of this treatment are the high costs and the insurance coverage issue. Oral and topical testosterone medications also seem to be effective for primary hypogonadism; however, they are slightly inferior to nasal sprays and often not covered by insurance. Insurance-covered TRT options for primary hypogonadism are often limited to injectable testosterone cypionate or enanthate. The longer-acting ester undecanoate is also often not covered. These injectable testosterone medications are far from optimal for maintaining fertility and will often cause a harsh shutdown of sperm production, even if used with ancillary drugs such as tamoxifen. Also, in the case of injectable testosterone medications, before initiating TRT, it is recommended to freeze sperm at a sperm bank or fertility clinic. Lastly, injectable TRT should be administered in cycles and discontinued after 3 months, followed by a post-TRT recovery cycle, in order to avoid chronic shutdowns of the HPGA. In the case of primary hypogonadism, post-cycle therapy might not be effective in restoring HPGA functions; therefore, freezing sperm is especially important in these individuals before starting TRT. In our opinion, hypogonadal men seeking treatment without side effects on fertility should first consider HCG or clomiphene treatment. If these options do not improve their situation, a short-acting form such as a nasal spray or oral testosterone should be considered. The addition of HCG, SERMs, or AIs to short-acting T treatment is also an option in case the sperm count declines. Long-acting injectable T treatment options, even in combination with HCG, SERMs, or AIs, involve much greater risks of developing infertility in TRT patients.
